# ﻿Two new species of the genus *Narynia* (Collembola, Isotomidae) from China

**DOI:** 10.3897/zookeys.1240.141871

**Published:** 2025-06-03

**Authors:** Cheng-Wang Huang

**Affiliations:** 1 Shanghai Entomological Museum, CAS Center for Excellence in Molecular Plant Sciences, Chinese Academy of Sciences, Shanghai, 200032, China CAS Center for Excellence in Molecular Plant Sciences, Chinese Academy of Sciences Shanghai China

**Keywords:** Continental Asia, identification key, *Proisotoma* complex, springtails, taxonomy

## Abstract

Two new species of the genus *Narynia* Martynova from China are described: *Naryniaalxaica***sp. nov.** from the western Inner Mongolia Autonomous Region, northern China and *Naryniaritongensis***sp. nov.** from Xizang Province, southwestern China. The two new species can be separated from known species by the length ratio (lateral vs. medial) and location of sensilla on abdominal segment V. An identification key to the species of the genus *Narynia* is given.

## ﻿Introduction

*Narynia* Martynova, 1967 is a small genus of Anurophorinae, which has a complete furca with a massive mucro, no anterior chaetae on the manubrium and a cluster of 10 conspicuous erect horizontal chaetae at the tip of the abdomen (Martynova 1967; Potapov 2001; Huang et al. 2010). Furthermore, the genus belongs to the *Proisotoma* complex, which has the following characteristics: reduction of furca variable, none or few anterior chaetae on the manubrium, and the two last abdominal segments clearly separated (Potapov et al. 2006; 2009, 2015, 2021; Huang and Potapov 2012; Potapov and Bogomolov 2016). At present, *Narynia* has four known species and primarily distributed across parts of the continental region of Inner Asia (Martynova and Tshelnokov 1975; Martynova 1981). *Narynialuanae* Huang, Potapov & Gao, 2010 is the only known species found in China (Northwest, Ningxia Province). In the present paper, two new species are described from China: *Naryniaalxaica* sp. nov. from Alxa Left Banner, Alxa League, western Inner Mongolia Autonomous Region, northern China and *Naryniaritongensis* sp. nov. from Ritong, Qamdo, Xizang Province, southwestern China.

## ﻿Material, methods and abbreviations

Samples were collected from the western Inner Mongolia Autonomous Region and Xizang Province of China. Collembola were extracted by Tullgren funnels and stored in 75% ethanol. Later, specimens were mounted on slides using Hoyer’s solution and dried in a drying box at 50–55 °C for five days. The specimens were examined by a Zeiss compound microscope Lab.A1. The figures were drawn using Nikon Microscopic Imaging System and improved with Photoshop CS5 (Adobe Inc.).

Type specimens of the new species are deposited in the Shanghai Entomological Museum, CAS Center for Excellence in Molecular Plant Sciences, Shanghai, China.

### ﻿Abbreviation used in this study

**Abd.I–VI** Abdominal segments I–VI

**accp1–4** p-row accessorial tergal sensilla 1–4 on abdominal segment V

**Ant.1–4** antennal segments 1–4

**bms** basal microsensillum on antennal segments

**CAS** Chinese Academy of Sciences

**Md, Mdl, Ml** dorsal, dorsolateral, lateral macrochaetae

**ms** microsensillum

**PAO** postantennal organ

**s** sensillum

**SEM** Shanghai Entomological Museum

**Th.I–III** thoracic segments I–III

**Ti.1–3** tibiotarsi of legs 1–3

**VT** ventral tube

## ﻿Results

### ﻿ Narynia alxaica sp. nov.

Taxon classificationAnimaliaCollembolaIsotomidae

69C2CE12-F7A3-5C5F-8BFA-E2161DB28876

https://zoobank.org/C52921F3-AB37-4156-B551-CBBBAADCAC95

Figs 1A–E , 2A

#### Type material.

***Holotype female*** • N China, W Inner Mongolia Autonomous Region, Alxa League, Alxa Left Banner, Yaoba, Qianggang range; mixed coniferous broad-leaved forest, moist sandy soil; 3 Aug. 2010; Cheng-Wang Huang and Yun Bu leg. ***Paratypes*** • N China, four females, four subadult females and subadult male; W Inner Mongolia Autonomous Region, Alxa League, Alxa Left Banner, Yaoba, Xiazi ravine; moist humus soil in the forest; 4 Aug. 2010; Cheng-Wang Huang and Yun Bu leg. Holotype and paratypes deposited in SEM.

#### Description.

Size about 1.2 mm. Colour greyish. Integument appears smooth, slightly polygonal under high magnification, without secondary granulation. All body chaetae smooth. Longest macrochaeta on Abd.IV about 0.3 times as long as tergite length.

**Figure 1.**
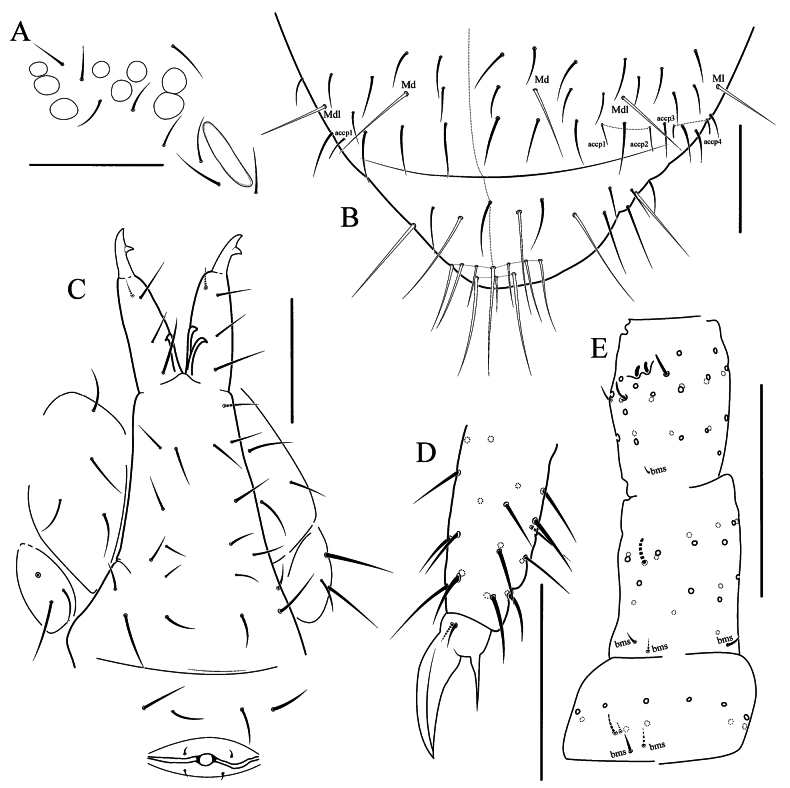
*Naryniaalxaica* sp. nov. **A**PAO and ommatidia **B** Abd.V–VI, dorsal view **C** furcal area and female genital plate **D** tibiotarsus and claw of Leg 3 **E** Ant.1–3, dorsal view. Scale bars: 50 μm.

Antennal length 0.8–0.9 times as long as head. Ant.1 with 11 ordinary chaetae, 2 bms (dorsal and ventral) and 2 ventral sensilla, the largest twice as long as the shortest. Ant.2 with 18 ordinary chaetae, 3 bms (dorsal, lateral and ventral) and 1 sensillum. Ant.3 with 24 ordinary chaetae, 1 bms and 1 lateral sensillum. Ant.3 organ with 2 inner sensilla (small sensory rods) and 2 guard sensilla (Fig. 1E). Ant.4 with numerous weakly differentiated sensilla, subapical microsensillum and organite present. Apical bulb absent. Ratio of Ant.1: 2: 3: 4 approximately 1: 1.4: 1.4: 2.4.

Ommatidia 8+8, G and H smaller, about 0.6 times as long as the diameter of ommatidium A (Fig. 1A). PAO broad, about 2.6 times as long as the adjacent ommatidium and about 1.0 times as long as width of Ant.1 and 1.1 times as long as inner unguis length, with 2 posterior chaetae. Maxillary palp bifurcate, with 4 sublobal hairs and 1 basal chaeta. Four prelabral chaetae, labral formula as 554. Labium with all papillae A–E present, papilla E with 7 guards, 3 proximal chaetae. Basomedial field of labium with 4 chaetae. Ventral side of head with 3+3 postlabial chaetae.

**Figure 2.**
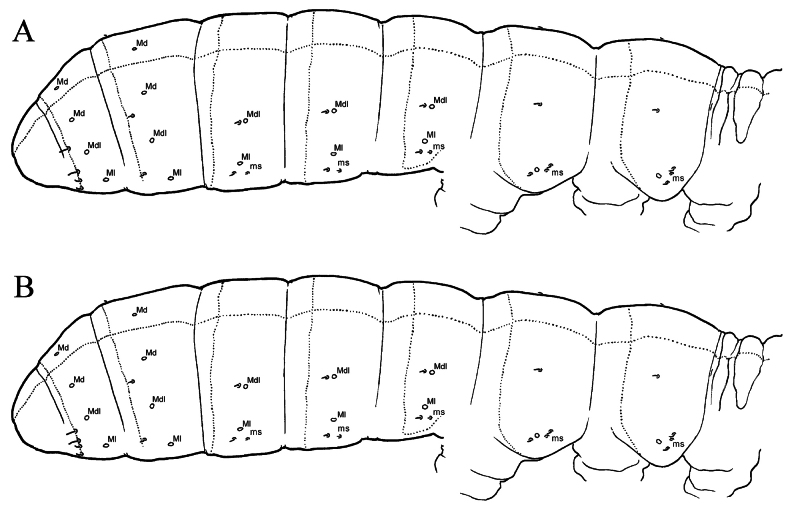
Sensillar chaetotaxy of *Narynia***A***Naryniaalxaica* sp. nov. **B***Naryniaritongensis* sp. nov.

Number of axial chaetae on each side of Th.II–Abd.V: (5)6,4/3,3,3,5(4 or 6),3, sometimes with 1 chaeta on center line of Abd.IV. Dorsal chaetotaxy of Abd.V–VI as in Fig. 1B. Macrochaetae smooth, 1,1/2,2,2,3,3 in number. On Abd.I–II Mdl shorter than Ml, on Abd.V Md about 0.6 times as long as tergite length. Macrosensilla on body short and slightly broadened, easily distinguished from ordinary chaetae. Sensillar formula as 33/22224 (Fig. 2A). On Abd.I–III macrosensilla positioned just posterior to Mdl. Abd.V with 2+2 well-marked, short lateral sensilla (accp3 and 4) and 2+2 medial sensilla (accp1 and 2), a little thinner and about 1.9 times as long as lateral sensilla. Microsensillar formula as 11/11100. Thorax without ventral chaetae. Abd.VI with 10 erect horizontal chaetae at tip.

Claw without inner and lateral teeth. Empodial appendage about half as long as unguis, rather thin. Ti.1–3 with 21, 21, 22 chaetae respectively, without clavate tenent hairs. Two inner chaetae on Ti.3 much thinner than others (Fig. 1D). VT with 4+4 laterodistal and 4 posterior chaetae, no anterior chaetae. Tenaculum with 3+3 teeth and 1 chaeta. Anterior furcal subcoxa with 4(3 or 5) chaetae, posterior subcoxa with 3(2 or 4) chaetae. Manubrium without anterior chaetae, posterior with 3+3 laterobasal, 2+2 in distal transversal row, and 4+(3)4 in central part. Dens with 1 anterior and 3 posterior chaetae (Fig. 1C). Mucro massive, bidentate. Ratio of manubrium: dens: mucro = 6.6: 2.5: 1. Female genital plate as in Fig. 1C.

#### Remarks.

*Naryniaalxaica* sp. nov. is similar to *Naryniakolimiensis* Martynova, 1981 and *N.ritongensis* sp. nov. They have the same location of the medial sensilla on Abd.I–III, no ventral chaetae on Th.III and the same chaetotaxy of the dens. However, they can easily be distinguished from each other by the location of the medial sensilla on Abd.V. The main differences between the three species are described in the Remarks of *N.ritongensis* sp. nov.

#### Distribution.

Known only from the type locality, the foothills of Helan Mountain.

#### Etymology.

The new species is named after the type locality.

### ﻿ Narynia ritongensis sp. nov.

Taxon classificationAnimaliaCollembolaIsotomidae

ACEB3EFE-8D7F-5E6A-BB49-7D4B73652535

https://zoobank.org/BB5BC1A5-E2C7-4497-A723-9D1038ECF0BB

Figs 2B , 3A–F

#### Type material.

***Holotype female*** • SW China; Xizang Province, Qamdo, Ritong; 6 Aug. 2009; Wan-Jun Chen leg. ***Paratypes*** • female and subadult male; same data as holotype. Holotype and paratypes deposited in SEM.

#### Description.

Size 0.9–1.0 mm. Colour greyish. Integument appears smooth, slightly polygonal under high magnification, without secondary granulation. All body chaetae smooth. Longest macrochaeta on Abd.IV about 0.3 times as long as tergite length.

**Figure 3.**
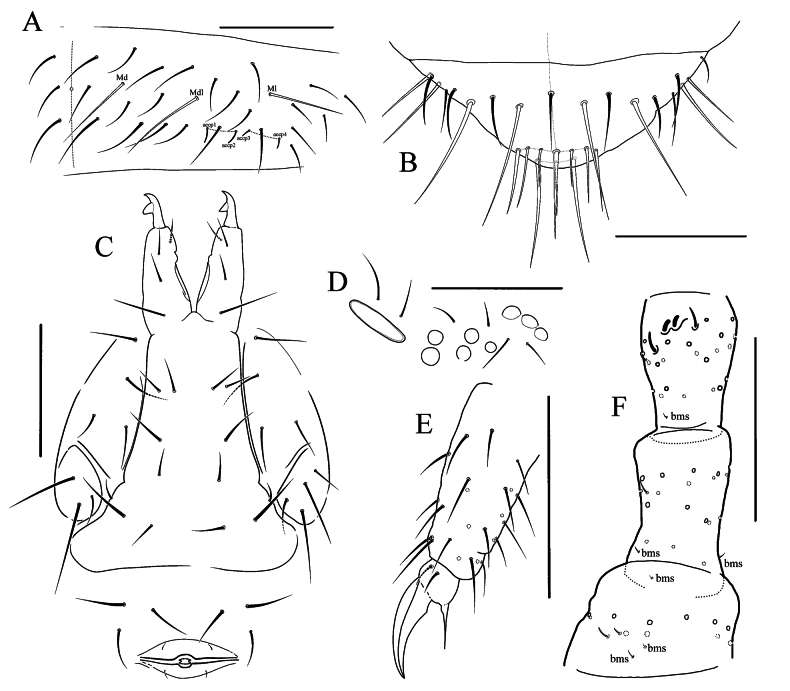
*Naryniaritongensis* sp. nov. **A** dorsal chaetotaxy of Abd.V **B** Abd.VI, dorsal view **C** furcal area and female genital plate **D**PAO and ommatidia **E** tibiotarsus and claw of Leg 3 **F** Ant.1–3, dorsal view. Scale bars: 50 μm.

Antennal length 0.8–1.0 times as long as head. Ant.1 with 11 ordinary chaetae, 2 bms (dorsal and ventral) and 2 ventral sensilla, the largest twice as long as the shortest. Ant.2 with 18 ordinary chaetae, 3 bms (dorsal, lateral and ventral) and 1 sensillum. Ant.3 with 22 ordinary chaetae, 1 bms and 1 lateral sensillum. Ant.3 organ with 2 inner sensilla (small sensory rods) and 2 guard sensilla (Fig. 3F). Ant.4 with numerous weakly differentiated sensilla, subapical microsensillum and organite present. Apical bulb absent. Ratio of Ant.1: 2: 3: 4 approximately 1: 1.3: 1.5: 2.2.

Ommatidia 8+8, G and H smaller, about 0.6 times as long as the diameter of ommatidium A (Fig. 3D). PAO broad, about 3.4 times as long as the adjacent ommatidium and about 0.9 times as long as width of Ant.1 and 1.2 times as long as inner unguis length, with 2 posterior chaetae. Maxillary palp bifurcate, with 4 sublobal hairs and 1 basal chaeta. Four prelabral chaetae, labral formula as 554. Labium with all papillae A–E present, papilla E with 7 guards, 3 proximal chaetae. Basomedial field of labium with 4 chaetae. Ventral side of head with 3+(1)3 postlabial chaetae.

Number of axial chaetae on each side of Th.II–Abd.V: 6,5/3,3,3,4–5,3, sometimes with 1 chaeta on center line of Abd.IV or V. Dorsal chaetotaxy of Abd.V–VI as in Fig. 3A, B. Macrochaetae smooth, 1,1/2,2,2,3,3 in number. On Abd.I–III Mdl shorter than Ml, on Abd.V Md about 0.6 times as long as tergite length. Macrosensilla on body short and slightly broadened, easily distinguished from ordinary chaetae. Sensillar formula as 33/22224 (Fig. 2B). On Abd.I–III macrosensilla positioned just posterior to Mdl. Abd.V with 2+2 well-marked, short lateral sensilla (accp3 and 4) and 2+2 medial sensilla (accp1 and 2), a little thinner and about 1.6 times as long as lateral sensilla. Microsensillar formula as 11/11100. Thorax without ventral chaetae. Abd.VI with 10 erect horizontal chaetae at tip.

Claw without inner and lateral teeth. Empodial appendage about half as long as unguis, rather thin. Ti.1–3 with 21, 21, and 24 chaetae, respectively, without clavate tenent hairs. Two inner chaetae on Ti.3 much thinner than others (Fig. 3E). VT with 4+4 laterodistal and 4 posterior chaetae, no anterior chaetae. Tenaculum with 3+3 teeth and 1 chaeta. Anterior furcal subcoxa with (3)4 chaetae, posterior subcoxa with 3(4) chaetae. Manubrium without anterior chaetae, posterior with 3+3 laterobasal, 2+2 in distal transversal row, and 2+2 in central part. Dens with 1 anterior and 3 posterior chaetae, basal chaeta on posterior side slightly larger (Fig. 3C). Mucro massive, bidentate. Ratio of manubrium: dens: mucro = 6.5: 2.5: 1. Female genital plate as in Fig. 3C.

#### Remarks.

*Naryniaritongensis* sp. nov. is similar to *N.alxaica* sp. nov. and *N.kolimiensis* by the location of medial sensilla on Abd.I–III, no ventral chaetae on Th.III and the same chaetotaxy of the dens. Although *N.luanae* has 1+1 ventral chaetae on Th.III, this species also shares several characteristics with the other three species. The main similarities and differences between the four species of *Narynia* are listed in Table 1.

**Table 1.** Diagnostic characters of *Narynia* species with dens of three posterior chaetae.

Character	*N.kolimiensis*	*N.luanae*	*N.alxaica* sp. nov.	*N.ritongensis* sp. nov.
Medial sensilla on Abd.I–III	posterior to Mdl	posterior to Mdl	posterior to Mdl	posterior to Mdl
Anterior/posterior chaetae on dens	1/3	1/3	1/3	1/3
Ventral chaetae on Th.III	0+0	1+1	0+0	0+0
Microsensilla on Th.III	interior to lateral s	between anterior and posterior s	between anterior and posterior s	between anterior and posterior s
Ratio lateral sensilla: medial sensilla on Abd.V	1: 1	1: 1.6–2.0	1: 1.6	1: 1.9
Location of accp1 on Abd.V	between Md and Mdl	between Md and Mdl	between Md and Mdl	between Mdl and Ml
Location of accp2 on Abd.V	between Md and Mdl	posterior to Mdl	between Mdl and Ml	between Mdl and Ml
Chaetae on Ti.3	24–25	22	22	24
Teeth on tenaculum	4+4	3+3	3+3	3+3
Chaetae on anterior furcal subcoxa	4	(4)5	4(3 or 5)	(3)4
Posterior chaetae on manubrium	8+8–9+9	(6)8–9+(6)8–9	9+(8)9	7+7
Ratio dens: mucro	2.2: 1	2.1–2.5: 1	2.5: 1	2.5: 1

#### Distribution.

Known only from the type locality.

#### Etymology.

The new species is named after the type locality.

### ﻿Key to species of the genus *Narynia*

**Table:** 

1	Th.III with ventral chaetae	**2**
–	Th.III without ventral chaetae	**4**
2	Abd.V with medial sensilla at most twice as long as lateral sensilla. Dens with at most 4 posterior chaetae	**3**
–	Abd.V with medial sensilla long, thin, 4 times longer than lateral sensilla. Dens with 5 posterior chaetae	***Naryniasetosa* Martynova, 1967**
3	Medial sensilla on Abd.I–III between Mdl and Ml. Dens with 4 posterior chaetae	***Naryniaandreevae* Martynova, 1975**
–	Medial sensilla on Abd.I–III slightly posterior to Mdl. Dens with 3 posterior chaetae	***N.luanae* Huang, Potapov & Gao, 2010**
4	Abd.V with medial sensilla long, thin, at least 1.6 times as long as lateral sensilla. Medial sensilla accp2 between Mdl and Ml	**5**
–	Abd.V with medial sensilla as long as lateral sensilla. Medial sensilla accp2 between Md and Mdl	***N.kolimiensis* Martynova, 1981**
5	Ti.3 with 22 chaetae. Medial sensilla accp1 on Abd.V between Md and Md	***N.alxaica* sp. nov.**
–	Ti.3 with 24 chaetae. Medial sensilla accp1 on Abd.V between Mdl and Ml	***N.ritongensis* sp. nov.**

## Supplementary Material

XML Treatment for Narynia alxaica

XML Treatment for Narynia ritongensis

## ﻿Additional information

### Author ORCIDs

Cheng-Wang Huang https://orcid.org/0000-0002-8666-7783

### Data availability

All of the data that support the findings of this study are available in the main text.

## References

[B1] Huang CW Potapov M (2012) Taxonomy of the *Proisotoma* complex. IV. Notes on chaetotaxy of femur and description of new species of *Scutisotoma* and *Weberacantha* from Asia. Zootaxa 3333: 38–49. 10.11646/zootaxa.3333.1.3

[B2] Huang CW Potapov M Gao Y (2010) Taxonomy of the *Proisotoma* complex. III. A revision of the genus *Narynia* (Collembola: Isotomidae) with a description of a new species from China. Zootaxa 2410: 45–52. 10.11646/zootaxa.2410.1.3

[B3] Martynova EF (1967) Materials on fauna of springtails (Collembola) of middle Asia. Izvestiya otdeleniya biologicheckikh nauk AN Tadjikskoi SSR 28: 32–46. [In Russian]

[B4] Martynova EF (1981) A new genus and new species of springtails (Collembola) from East Siberia. Zoologichesky Zhurnal 60(1): 151–157. [In Russian]

[B5] Martynova EF Tshelnokov VG (1975) Collembola from the East Pamirs. II. The family Isotomidae. Entomologicheskoe Obozrenie 4: 787–793. [In Russian]

[B6] Potapov M (2001) Synopses on Palaearctic Collembola. Volume 3. Isotomidae. Abhandlungen und Berichte des Naturkundemuseums, Görlitz, 603 pp.

[B7] Potapov M Bogomolov M (2016) Taxonomy of the *Proisotoma* complex. VI. Mobile forms of *Proisotoma**s.str.* with the description of a new species from East Siberia (Collembola: Isotomidae). Zootaxa 4088(2): 257–267. 10.11646/zootaxa.4088.2.7 27394339

[B8] Potapov M Babenko A Fjellberg A (2006) Taxonomy of the *Proisotoma* complex. Redefinition of genera and description of new species of *Scutisotoma* and *Weberacantha* (Collembola, Isotomidae). Zootaxa 1382: 1–74. 10.11646/zootaxa.1382.1.1

[B9] Potapov M Babenko A Fjellberg A Greenslade P (2009) Taxonomy of the *Proisotoma* complex. II. A revision of the genus *Subisotoma* and a description of *Isotopenola* gen. nov. (Collembola: Isotomidae). Zootaxa 2314: 1–40. 10.11646/zootaxa.2314.1.1

[B10] Potapov M Kahrarian M Deharveng L Shayanmehr M (2015) Taxonomy of the *Proisotoma* complex. V. Sexually dimorphic *Ephemerotoma* gen. nov. (Collembola: Isotomidae). Zootaxa 4052(3): 345–358. 10.11646/zootaxa.4052.3.4 26701434

[B11] Potapov M Deharveng L Janion-Scheepers C (2021) Taxonomy of the *Proisotoma* complex. VI. Rediscovery of the genus *Bagnallella* Salmon, 1951 and epitoky in *Bagnallelladavidi* (Barra, 2001), comb. nov. from South Africa. ZooKeys 1072: 167–186. 10.3897/zookeys.1072.71307 PMC8632875 34899011

